# Divergent evolution of two corticotropin-releasing hormone (CRH) genes in teleost fishes

**DOI:** 10.3389/fnins.2015.00365

**Published:** 2015-10-13

**Authors:** Brian P. Grone, Karen P. Maruska

**Affiliations:** ^1^Department of Neurological Surgery, University of California, San FranciscoSan Francisco, CA, USA; ^2^Department of Biological Sciences, Louisiana State UniversityBaton Rouge, LA, USA

**Keywords:** corticotropin-releasing factor (CRF), genome duplication, subfunctionalization, cichlid, zebrafish, spotted gar, brain, retina

## Abstract

Genome duplication, thought to have happened twice early in vertebrate evolution and a third time in teleost fishes, gives rise to gene paralogs that can evolve subfunctions or neofunctions via sequence and regulatory changes. To explore the evolution and functions of corticotropin-releasing hormone (CRH), we searched sequenced teleost genomes for CRH paralogs. Our phylogenetic and synteny analyses indicate that two CRH genes, *crha* and *crhb*, evolved via duplication of *crh1* early in the teleost lineage. We examined the expression of *crha* and *crhb* in two teleost species from different orders: an African cichlid, Burton's mouthbrooder, (*Astatotilapia burtoni*; Order Perciformes) and zebrafish (*Danio rerio*; Order Cypriniformes). Furthermore, we compared expression of the teleost *crha* and *crhb* genes with the *crh1* gene of an outgroup to the teleost clade: the spotted gar (*Lepisosteus oculatus*). *In situ* hybridization for *crha* and *crhb* mRNA in brains and eyes revealed distinct expression patterns for *crha* in different teleost species. In the cichlid, *crha* mRNA was found in the retina but not in the brain. In zebrafish, however, *crha* mRNA was not found in the retina, but was detected in the brain, restricted to the ventral hypothalamus. Spotted gar *crh1* was found in the retina as well as the brain, suggesting that the ancestor of teleost fishes likely had a *crh1* gene expressed in both retina and brain. Thus, genome duplication may have freed *crha* from constraints, allowing it to evolve distinct sequences, expression patterns, and likely unique functions in different lineages.

## Introduction

Corticotropin-releasing hormone (CRH), also referred to as corticotropin-releasing factor (CRF) (Hauger et al., 2003), is known primarily for its central role in regulating neuroendocrine responses to stressors via release from the hypothalamus (Vale et al., 1981; Korosi and Baram, 2008). Yet CRH and other neuroendocrine releasing hormones are also expressed more broadly throughout the brain (Swanson et al., 1983). CRH immunoreactivity has also been found in retinal amacrine cells, including displaced amacrine cells, and ganglion cells in several species, i.e., chicken (Kiyama et al., 1984), rat (Skofitsch and Jacobowitz, 1984), and turtle (Williamson and Eldred, 1989). CRH-expressing neurons play diverse roles throughout the brain and in the retina, but many of these have not been fully explored (Kovács, 2013).

Many gene families in vertebrates were generated during two rounds of whole-genome duplication early in vertebrate evolution (Ohno, 1970; Abi-Rached et al., 2002; Dehal and Boore, 2005). Recently, we found that an ancestral vertebrate *CRH* gene experienced a duplication early in vertebrate evolution, giving rise to two ohnologs—paralogous genes that originated via whole-genome duplication—*CRH1* and *CRH2* (Grone and Maruska, 2015). We found both *CRH1* and *CRH2* in many groups of vertebrates, including ray-finned fishes, reptiles, birds, and mammals (Grone and Maruska, 2015), in addition to the elephant shark (*Callorhinchus milii*), where it was originally reported (Nock et al., 2011). Although *CRH1* and *CRH2* genes share sequence homology, CRH1 protein sequences are much more conserved compared to the highly variable CRH2 sequence, suggesting that the CRH1 protein retained essential structure and functions. Furthermore, expression of *crh2* mRNA in spotted gar brain is much more restricted compared to *crh1* mRNA (Grone and Maruska, 2015). Spotted gar *crh1* and *crh2* expression has not, however, been examined in the retina. Indeed, expression of *crh2* has not been examined in the retina of any species.

No identifiable *crh2* gene ortholog is found in any sequenced teleost species, suggesting that *crh2* was lost early in teleost evolution (Grone and Maruska, 2015). Teleost fishes, however, experienced an additional (third) whole genome duplication (WG3) prior to their ecological and evolutionary radiation (Christoffels et al., 2004; Hoegg et al., 2004; Jaillon et al., 2004; Amores et al., 2011). Many teleost genes are thus present in duplicate compared to their mammalian homologs. In the course of describing *crh1* and *crh2*, we also showed that many teleost species' genomes contain two *crh1* orthologs: *crha* and *crhb* (Grone and Maruska, 2015). These two teleostean *crh* ohnologs encode different predicted 41-amino-acid processed peptides. Only *crhb* has been studied, while *crha* has gone unremarked. In fact, until recently, only one *crh* gene was thought to exist in zebrafish and many other teleosts (Chandrasekar et al., 2007; Lovejoy et al., 2014).

Genome sequences are now available for several teleosts, including zebrafish (Howe et al., 2013), medaka (Kasahara et al., 2007), three-spined stickleback (Jones et al., 2012), Atlantic cod (Star et al., 2011), rainbow trout (Berthelot et al., 2014), and several African cichlid species (Brawand et al., 2014). The synteny, sequences, and phylogenetic relationships of the duplicated CRH genes in these species have not been previously studied, and expression patterns of *crha* have not been reported for any species. Comparing duplicated genes in teleosts to the orthologs in spotted gar, a primitive non-teleost ray-finned fish, can generate insights regarding diverse evolutionary processes including gene duplication, gene loss, sequence evolution, and regulatory changes (Braasch et al., 2015; Gehrke et al., 2015).

In the present study, we first employed comparative genomic and phylogenetic analyses to identify the evolutionary relationships of the teleost CRH genes, *crha* and *crhb*, to other CRH-family peptides. We then used *in situ* hybridization in the brain and retina of two teleosts, the zebrafish, *Danio rerio*, and an African cichlid fish, *Astatotilapia burtoni*, as well as the spotted gar, *Lepisosteus oculatus*, to characterize and analyze expression patterns of CRH genes. Our analyses reveal a diverse range of evolutionary outcomes following CRH gene duplication, with potential functional implications for stress-regulating systems and neuromodulation of brain and retinal function across vertebrates.

## Materials and methods

### Animals

Juvenile *Lepisosteus oculatus* were purchased from Rainforest International (Bloomington, IN) or caught from the Atchafalaya Basin, LA. *Danio rerio* were purchased from Arizona Aquatic Gardens (Oro Valley, AZ). *Astatotilapia burtoni* (Fernald, 1977) originally derived from a wild-caught population were maintained at LSU. Juvenile *Lepisosteus oculatus* (*n* = 5; Standard Length (SL) = 69.6 ± 12.4 mm) (mean±sd), adult *Danio rerio* (*n* = 3 males, 2 females; *SL* = 25.8 ± 1.3 mm), and adult *Astatotilapia burtoni* (*n* = 5 males; *SL* = 45.4 ± 5.1 mm) were used for the *in situ* hybridization (ISH) experiments. All experiments were performed in accordance with the recommendations and guidelines stated in the National Institutes of Health (NIH) Guide for the Care and Use of Laboratory Animals, 2011. The protocol was approved by the Institutional Animal Care and Use Committee (IACUC) at Louisiana State University, Baton Rouge, LA.

### Sequence analysis

Throughout this paper, we use standard gene nomenclature. For fishes, gene symbols are italicized and protein symbols are capitalized. For other vertebrates, human conventions are used: gene symbols in all capitals and italicized, protein symbols in all capitals.

To determine the phylogenetic relationships among fish CRH gene family members, sequences for CRH-family genes were located by searches in Ensembl for spotted gar (*Lepisosteus oculatus*), Atlantic cod (*Gadus morhua*), and zebrafish (*Danio rerio*), and in GenBank for Burton's mouthbrooder (*Astatotilapia burtoni*), medaka (*Oryzias latipes*), rainbow smelt (*Osmerus mordax*), Atlantic salmon (*Salmo salar*), and bicolor damselfish (*Stegastes partitus*) (Table 1). Zebrafish urotensin 1 (*uts1*) sequence was retrieved from GenBank (NP_001025351.1). In Table 1, Ensembl identification numbers or GenBank accession numbers are listed, except for the case of *Gasterosteus aculeatus crha*, for which the position on a scaffold is given. ORFs for unannotated *crha* genes were predicted from genomic DNA using GENSCAN (Burge and Karlin, 1997). Predicted amino acid sequences were then generated from ORFs.

**Table 1:** **Sources for teleost CRH family genes described in this paper**.

**Teleost species**	**Common name**	**crha**	**crhb**
*Astatotilapia burtoni*	Burton's mouthbrooder	XM_005946397	EF363131
*Danio rerio*	Zebrafish	ENSDARG00000093401	ENSDARG00000027657
*Gadhus morhua*	Atlantic cod	ENSGMOG00000020152	ENSGMOG00000002107
*Gasterosteus aculeatus*	Three-spined stickleback	scaffold_120: 91757-91374	ENSGACG00000002971
*Oryzias latipes*	Medaka	ENSORLG00000006816	ENSORLG00000008503
*Osmerus mordax*	Rainbow smelt	BT075587.1	BT075567.1
*Salmo salar*	Atlantic salmon	crha1: EG786316 crha2: EG808138	crhb1: DY733166 crhb2: BT057824
*Stegastes partitus*	Bicolor damselfish	XM_008303879.1	XM_008288433.1
*Takifugu rubripes*	Fugu		ENSTRUT00000022109

All multiple sequence alignments were carried out using MAFFT (Katoh and Standley, 2013) in Geneious software version 5.1.7 (Kearse et al., 2012), with the following settings: Algorithm = E-INS-i; scoring matrix = BLOSUM62; gap-open penalty = 1.53.

MEGA5 identified the best amino acid substitution model as JTT+G+I (Tamura et al., 2011), according to comparisons of the Bayesian Information Criteria for 48 different amino acid models. Therefore, we used the Jones-Taylor-Thornton model (JTT) (Jones et al., 1992), with non-uniformity of evolutionary rates among sites modeled by estimating a discrete Gamma distribution (+G) and by assuming that certain sites are evolutionarily invariable (+I).

An unrooted Maximum Likelihood phylogenetic tree was created from the protein alignment using PhyML (Guindon and Gascuel, 2003) in Geneious software with the JTT+G+I model. The following parameters were used: Tree topology search: NNIs; Initial tree: BioNJ, Number of substitution rate categories: 4. The tree was checked via 1000 bootstrap iterations.

Gene synteny for teleost *crha and crhb* genes was compared to the syntenic region near spotted gar *crh1* using Genomicus (Louis et al., 2013).

### Colorimetric *in situ* hybridization

To localize *crha* and *crhb* mRNA in the brain and retina of Burton's mouthbrooder and zebrafish, and *crh1* and *crh2* in the brain and retina of spotted gar, we performed chromogen-based *in situ* hybridization with riboprobes on cryosectioned tissue. DNA templates for riboprobes were generated by PCR amplification (Platinum PCR SuperMix, Life Technologies) of genomic DNA from the corresponding species using commercially synthesized (Life Technologies) gene-specific primers (Table 2) and the following reaction conditions: 95°C for 1 min, 40 cycles of: (95°C for 15 s, 55°C for 15 s, 72°C for 1 min), and 72°C for 1 min. Purified PCR products (MinElute PCR Purification Kit, Qiagen) were then used as templates for the transcription reactions. Probes were designed to be complementary to the longest exon (exon 2) for each gene and were transcribed from the T3 polymerase transcription initiation sequence (aattaaccctcactaaaggg) that was added to the reverse (for anti-sense probes) or forward (for sense control probes) template primer. Digoxigenin (DIG)-labeled antisense and sense riboprobes for *L. oculatus crh1* and *crh2* (as described previously Grone and Maruska, 2015) and for *A. burtoni* and *D. rerio* genes, *crha* and *crhb*, were transcribed *in vitro* using T3 RNA Polymerase (Fermentas) and DIG RNA labeling mix (Roche Molecular Biochemicals), with RNAse Out (Life Technologies), and were then treated with Turbo DNAse (Ambion Inc.) and purified using illustra ProbeQuant G-50 Micro Columns (GE Healthcare). PCR products and final probes were checked on a 1% agarose gel after each step and verified to be single bands of the correct size (see Table 2 for probe sizes).

**Table 2:** **Primer sequences^a^ used to generate templates for the synthesis of riboprobes used for *in situ* hybridization**.

**Gene**	**Forward primer (5^′^ → 3^′^)**	**Reverse primer (5^′^ → 3^′^)**	**Probe length (bp)**
*L. oculatus crh1*	AGCTCAATTTCCTTGTTTCTACTGC	CATCATTTTTCTGTTGCTGTGCGC	479
*L. oculatus crh2*	GCCATGAGCAAGTTGCTTCTCCTG	CAGCTTTAGCCATTTCTAGGAACTC	400
*A. burtoni crha*	CAGACTGCCCTCTGAGATGAAGC	TCTTCTTCACTTTCCGAGGGTATCC	448
*A. burtoni crhb*	GACTCGAACTCTTTCCCATC	ATGAAGAACGGCATATCTCTAC	545
*D. rerio crha*	GGACGATTTAGGAAAATGAAGC	TGTTCATTTTTTAAATATAGG	238
*D. rerio crhb*	CCTCGCCACTTTTTGACATGAAGC	CTCTTGCACAACAAAAATTAATGG	904

a *T3 transcription initiation sequence (aattaaccctcactaaaggg) was added to the reverse primer (for anti-sense probes) or forward primer (for sense control probes)*.

Fish were anesthetized using ice water and decapitated. All solutions used for tissue preparation and ISH were made with RNAse-free 0.25-μm-filtered water. The top of the skull was removed to expose the brain, and the brain was fixed in the head overnight at 4°C with 4% paraformaldehyde (PFA) made in phosphate buffered saline (PBS, pH 7.2). Heads were then transferred to PBS and washed overnight at 4°C. Brains and left eyes were then removed from the heads (lenses were removed from the eyes by making a small slit in the dorsal portion of the eye) and cryoprotected in 30% sucrose at 4°C for 1–3 days until sectioning.

Brains and eyes were mounted in Tissue-Tek OCT (Sakura) and sectioned at 20 μm in the transverse plane with a cryostat (Thermo, Cryostar NX50). Sections from each brain and eye were collected onto 2–4 sets of alternate charged slides (VWR, Superfrost Plus), dried at room temperature for 2 days, and then stored at −80°C prior to use in ISH. Using an ImmEdge Hydrophobic Barrier Pen (Vector Laboratories), a hydrophobic barrier was drawn on the edges of each slide and allowed to dry at room temperature (30 min). Sections were rehydrated with 3 × 5 min washes in PBS, fixed with 4% PFA for 20 min at room temperature, washed 2 × 5 min with PBS, and permeabilized with proteinase K (10 μg/ml) for 10 min at room temperature. Sections were then washed 1 × 5 min with PBS at room temperature, postfixed with 4% PFA for 15 min at room temperature, and washed with 2 × 5-min PBS washes and one short rinse in water. Sections were treated with 0.25% acetic anhydride in 0.1 M triethanolamine-HCl, pH 8.0, which was mixed and vortexed immediately before use. Following acetic anhydride treatment, sections were washed 1 × 5 min with PBS at room temperature, and then incubated in pre-warmed hybridization buffer in a sealed humidified chamber to pre-hybridize at 60–65°C for 3 h. When pre-hybridization was complete, the hybridization solution was removed and replaced with hybridization buffer containing gene-specific probes, to generate multiple complete series of brain sections for each gene studied. Slides were covered with HybriSlip hybridization covers (Life Technologies) to help prevent evaporation and to distribute the probe evenly on the slide, and hybridization was carried out overnight (12–16 h) at 60–65°C in sealed humidified chambers. Sections were then washed 2 × 30 min at 60–65°C in pre-warmed 2X SSC in 50% Formamide with 0.1% Tween-20, 2 × 15 min at 60–65°C in 1:1 mixture of 2X SSC and maleic acid buffer with 0.1% Tween-20 (MABT), and 2 × 10 min at 60–65°C with MABT. Slides were then washed 2 × 10 min with MABT at room temperature, and non-specific binding was blocked by incubation in MABT with 2% bovine serum albumin (BSA) for 3 h at room temperature. Slides were then incubated with alkaline-phosphatase-conjugated anti-DIG Fab fragments (Roche) diluted 1:5000 in MABT 2% BSA overnight at 4°C in a sealed humidified chamber. Following antibody incubation, slides were washed with MABT (3 × 30 min) at room temperature, rinsed 2 × 5 min in alkaline phosphatase buffer at room temperature, and then developed with nitro blue tetrazolium/5-bromo-4-chloro-3-indolyl phosphate (NBT/BCIP) substrate (Roche) at 37°C in the dark for 1–3 h. Slides were then washed in PBS at room temperature to stop the reaction (3 × 5 min), fixed in 4% PFA for 10 min, washed in PBS (3 × 5 min), and coverslipped with Aqua-Mount media (Thermo Scientific).

For each species, staining with anti-sense probes was strong, specific (minimal background), and consistent across all individuals examined. To test for probe specificity, sense control probes were applied to one set of alternate sections (transverse, 20 μm) for each gene and run simultaneously with anti-sense probes applied to another set of alternate slides. None of these sense controls showed any labeling.

### Double fluorescent *in situ* hybridization (FISH)

Fluorescent probe preparation was similar to that described above except that fluorescein-labeled nucleotides (FITC-labeling mix, Roche) were incorporated into the nucleic acid sequence during the transcription reaction. Prior to hybridization, the double FISH protocol was also identical to that described above for chromogenic ISH. Hybridization for double FISH was performed by simultaneously applying two probes (DIG-labeled *crha* and fluorescein-labeled *crhb*) to the tissue followed by incubation at 60–65°C overnight in sealed chambers as described above. Slides were then washed with 2 × SSC: formamide with 0.1% tween-20 (2 × 30 min) at 60–65°C, followed by room temperature incubation in Tris-NaCl-Tween (TNT) buffer (2 × 10 min), quenching in TNT + 0.3% H_2_O_2_ (10 min), and rinsing in TNT buffer (3 × 5 min). Non-specific binding was then blocked with 0.5% blocking reagent (Roche) in TNT (1.5 h) followed by incubation with Anti-Fluorescein-POD Fab fragments (Roche; 1:250 dilution) overnight at 4°C. Slides were next rinsed with TNT buffer (4 × 10 min), incubated with Alexa Fluor 488 tyramide (2 h), washed with TNT buffer (2 × 5 min), quenched with TNT + 0.3% H_2_O_2_ (15 min), washed with TNT buffer (2 × 5 min) and blocked with 0.5% blocking reagent in TNT (1 h). Slides were then incubated with anti-DIG Fab fragments (Roche; 1:5000 dilution) for 2 h at room temperature, washed with TNT buffer (2 × 5 min), and developed with Fast Red (1–2 h at 37°C; Roche). Reactions were stopped by washing in TNT buffer (2 × 5 min), and slides were mounted with Fluorogel II containing DAPI nucleic acid counterstain (Electron Microscopy Services) and coverslipped.

### Imaging and analysis

To identify the expression patterns of genes in the brain and retina, DIG-labeled sections were imaged using brightfield illumination and FISH-labeled sections were imaged using fluorescence on a Nikon Eclipse Ni microscope. Photographs were taken with a color (Nikon DS-Fi2; for DIG-labeled) or monochrome (Nikon DS-Qi1Mc; for fluorescent-labeled) digital camera controlled by Nikon Elements software. Images were sharpened and adjusted for contrast, brightness, and levels as needed in Photoshop CS6 (Adobe Systems).

## Results

### Sequence analyses

**Figure 1:**
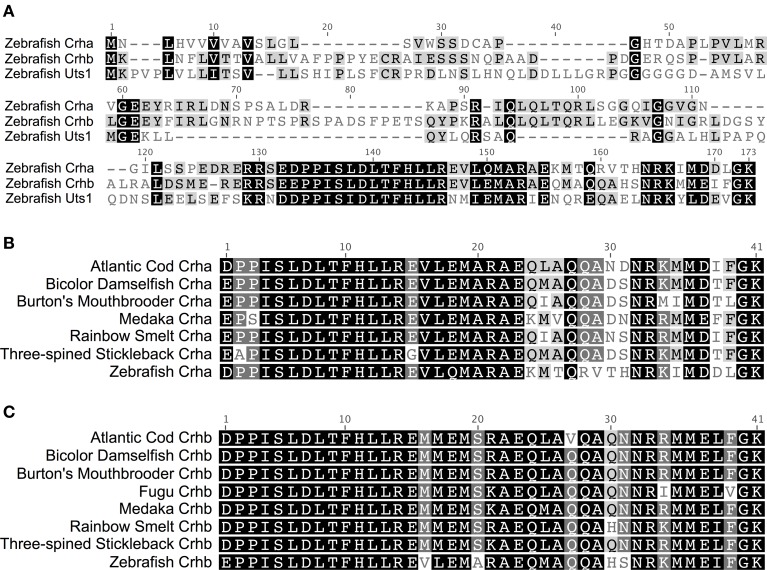
**Two CRH genes, *crha* and *crhb*, are conserved across diverse teleost species**. **(A)** Alignment of full-length translated CDS of zebrafish Crha, Crhb, and Uts1. Black, amino acids similar in all three sequences; Gray, similar to one corresponding residue; White, not similar to either corresponding residue. **(B)** Teleost Crha alignment. The predicted 41-amino-acid processed peptide sequences for Crha were aligned for seven teleost species. **(C)** Teleost Crhb alignment. The predicted 41-amino-acid processed peptide sequences for Crhb were aligned for eight teleost species, including fugu. Black, 100% of residues are similar across species; Dark Gray, 80–100% of residues are similar; Light Gray, 60–80% of residues are similar; White, less than 60% of residues are similar. Sequences were aligned using MAFFT software with the E-INS-i algorithm and the default settings. Amino acid similarity: Score Matrix, BLOSUM62; Threshold, 2.

In teleost fishes, the amino acid sequence of Crha is much more similar to Crhb than to Uts1 (Figure 1A). Zebrafish (*Danio rerio*) Crha and Crhb full-length peptide sequences share 45.1% pairwise identity, with 70.7% pairwise identity in the 41-amino-acid processed peptide region. Zebrafish Crha and Uts1, on the other hand, share only 29.2% pairwise identity of their full-length sequences and 56.1% pairwise identity in the 41-amino-acid processed peptide region.

Crha peptides have a higher degree of sequence variability across species than do Crhb peptides. The 7 Crha predicted processed 41-amino-acid peptide sequences we compared (Figure 1B) show on average 80.6% pairwise identity and 24 of 41 (58.5%) invariant amino acid residues. The 8 predicted Crhb processed 41-amino-acid peptide sequences we compared (Figure 1C) show on average 90.0% pairwise identity and 30 of 41 (73.2%) invariant amino acid residues.

The C-terminal region of the Crha peptides showed much greater sequence variability than the N-terminal portion. The 18 C-terminal amino acid residues have 64.8% average pairwise identity across the species examined, and only 6 of 18 (33.3%) are invariant across species. On the other hand, the 23 N-terminal amino acid residues have 93.0% average pairwise identity, and 18 of 23 (78.3%) are invariant across species.

Crhb, in contrast to Crha, had low sequence variability across species even in the C-terminal region. The 18 C-terminal amino acid residues have 85.3% average pairwise identity and 11 of 18 (61.1%) are invariant across species. In Crhb, the N-terminal 23 amino acid residues were less variable, with 93.3% average pairwise identity among species and 18 of 23 (78.3%) invariant residues across species.

No *crha* gene was identifiable in some teleost genomes. In particular, fugu (*Takifugu rubripes*) and pufferfish (*Tetraodon nigroviridis*) appear to have lost *crha*. A region of the tetraodon genome containing *trim55a* (Chromosome 15_random:114509–145684) did not contain an identifiable *crha* homolog. Similarly, BLAST searches of the tetraodon genome revealed no *crha*.

The Atlantic cod *crhb* gene is currently listed in Ensembl as a pseudogene (ENSGMOG00000002107), but Genscan predicts that it encodes a peptide with homology to Crhb. Further supporting its presence as a true gene, mRNA transcripts of this *crhb* gene in Atlantic cod have been identified from cDNA (GU828012.1).

### Synteny

**Figure 2:**
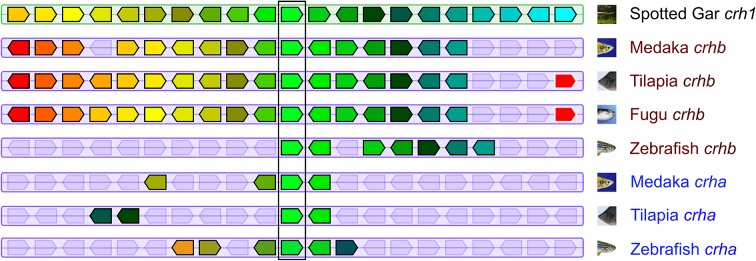
**Teleost *crha* and *crhb* genes share synteny with spotted gar *crh1***. The central pentagons (surrounded by a vertical rectangle) represent CRH genes. Spotted gar *crh1* is at the top center. Directly below are medaka, tilapia, fugu, and zebrafish *crhb* genes. Below these are medaka, tilapia, and zebrafish *crha* genes. For each CRH gene, 10 flanking genes on each side are represented by colored pentagons. Each color identifies a set of orthologous genes, and the pentagons point in the direction of transcription. Figure modified from Genomicus phyloview output.

Teleost *crha* and *crhb* genes are found on chromosomes adjacent to similar genes (Figure 2). In tilapia (*Oreochromis niloticus*), medaka, and fugu, *crhb* genes are in regions of more highly conserved synteny compared to *crha* genes. For example, of 20 genes adjacent to tilapia *crhb* (10 upstream and 10 downstream), 17 have homologs among the group of 30 genes adjacent to spotted gar *crh1* (15 upstream and 15 downstream). In contrast, only 3 of the 20 genes adjacent to tilapia *crha* have homologs among the group of 30 genes adjacent to spotted gar *crh1*.

### Phylogenetics

**Figure 3:**
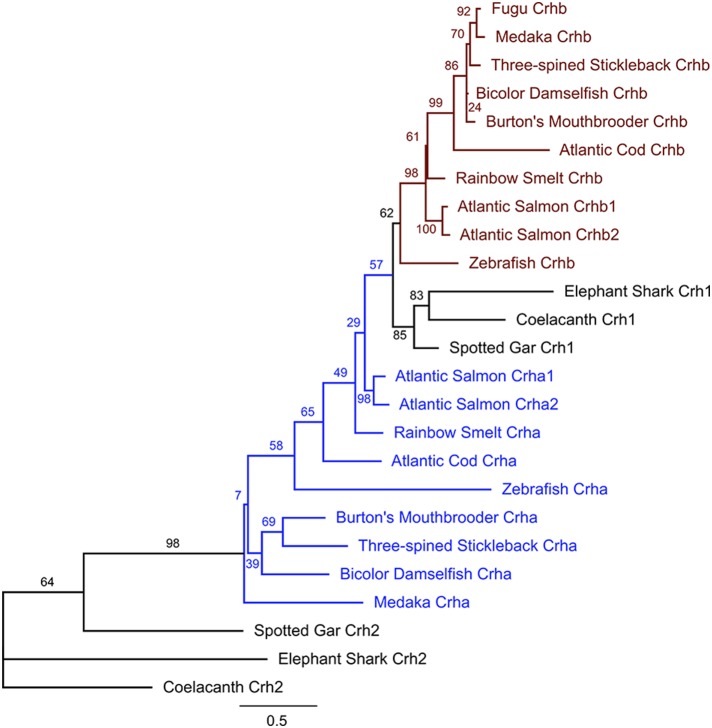
**Maximum likelihood phylogenetic tree of teleost Crha, Crhb, Crh1, and Crh2 based on predicted protein sequences**. Protein sequences translated from the ORFs of each gene were aligned using MAFFT software, and the unrooted tree was calculated using PhyML (see Materials and Methods for details). For each teleost species in the tree, all available CRH peptides were included. Nodes are labeled with % bootstrap support following 1000 bootstrap replicates. Branch length is proportional to number of substitutions per site and the scale bar indicates length of 0.5 substitutions/site.

Teleost Crhb and Crha sequences are more closely related to Crh1 than to Crh2 sequences from non-teleost fishes (Figure 3). Teleost Crhb protein sequences form a distinct clade (Figure 3). Teleost Crha protein sequences, on the other hand, are much more divergent in sequence and are recovered as a related grade in our tree. Predicted species relationships are reflected in the tree, as the spiny-rayed fishes (acanthomorpha), including cichlid, medaka, bicolor damselfish, fugu, and three-spined stickleback, form related groups for both Crha and Crhb. Sequences from the other fishes are more distantly related, as expected. Furthermore, as expected from the greater sequence variability of Crha, the branch length for Crha proteins is on average longer than for Crhb proteins.

### *crh* localization in the retina

**Figure 4:**
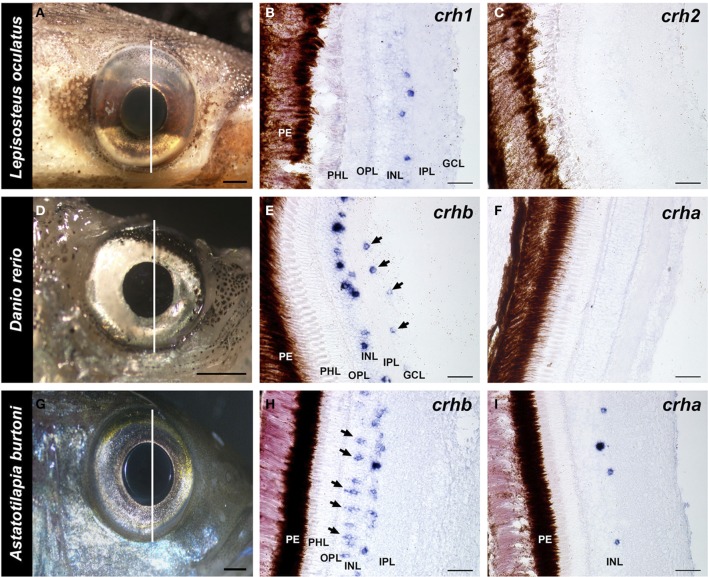
**Localization of *crh* forms in the retina of fishes**. Photographs of the eye of each species and adjacent 20 μm transverse sections of the retina reacted with each *crh* anti-sense probe are shown. **(A–C)** The spotted gar *Lepisosteus oculatus* shows *crh1* expression in the inner nuclear layer, but *crh2* is absent from the retina. **(D–F)** The zebrafish *Danio rerio* expresses *crhb* in the amacrine cell region of the inner nuclear and in the ganglion cell layer (arrows), but *crha* is absent from the retina. **(G–I)** The African cichlid fish *Astatotilapia burtoni* shows *crhb* label in two different cell types within the inner nuclear layer, amacrine and bipolar cells (arrows). *crha* was also found in the *A. burtoni* retina, but was restricted to the region of amacrine cells in the inner nuclear layer. GCL, ganglion cell layer; INL, inner nuclear layer; IPL, inner plexiform layer; OPL, outer plexiform layer; PHL, photoreceptor layer; PE, pigmented epithelium. Lines on **(A,D,G)** indicate the approximate position of the sections. Scale bars = 1 mm **(A,D,G)**; 25 μm **(B,C,E,F,H,I)**.

In the spotted gar retina, *crh1*-expressing cells were found exclusively in the inner nuclear layer (INL), near the border of the inner plexiform layer (IPL) (Figure 4B). Based on morphology and location, these are likely a type of amacrine cell. On the other hand, no *crh2* mRNA was found in the spotted gar retina (Figure 4C).

Both teleost species we examined showed expression of *crhb* in the retina, but expression patterns differed between the zebrafish and the cichlid (Figure 4). In the zebrafish, there were abundant *crhb*-expressing cells in the INL region where amacrine cells are found along the IPL border (Figure 4E). There were also scattered labeled cells in the ganglion cell layer (GCL) of the zebrafish retina, but it is unknown whether these are ganglion cells or displaced amacrine cells. In the cichlid, there were similarly abundant *crhb*-expressing cells in the amacrine cell region of the INL, but there were also abundant *crhb* cells found deeper in the INL at the border of the OPL (Figure 4H). Based on their location and morphology, these *crhb*-expressing cells in the cichlid are likely bipolar cells. In contrast to the zebrafish, *crhb* was not observed in the GCL of the cichlid retina.

**Figure 5:**
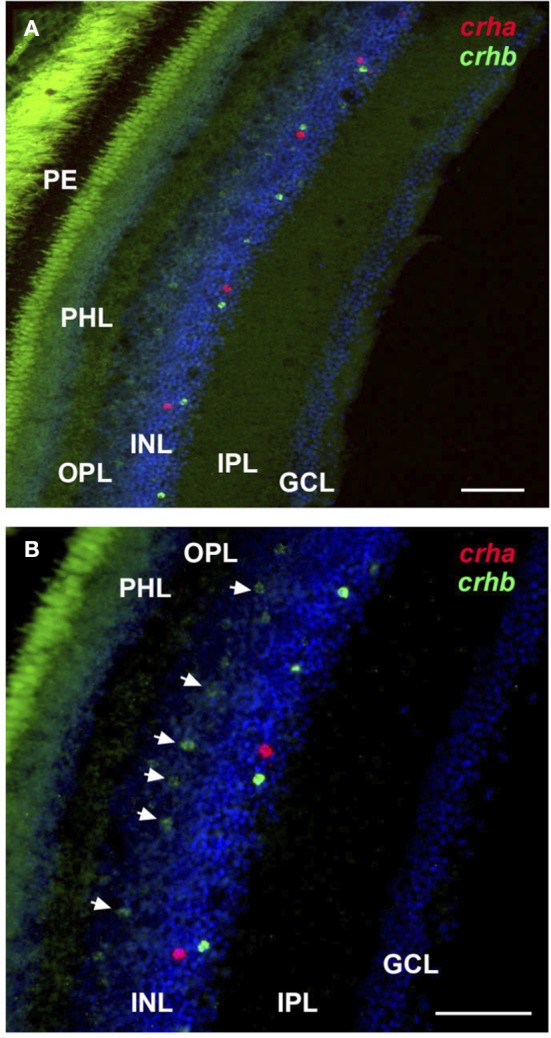
**Localization of *crha* and *crhb* in the retina of the cichlid fish *Astatotilapia burtoni***. Low **(A)** and high **(B)** magnification of the retina shows *crha* (red) and *crhb* (green) localized to the amacrine cell region of the inner nuclear layer at the border of the inner plexiform layer. Bipolar cells expressing *crhb* (arrows) are also found in the region of the inner nuclear layer that borders the outer plexiform layer. Transverse 20 μm sections from the same region indicated in Figure 4 are shown, and tissue was counterstained with DAPI (blue). GCL, ganglion cell layer; INL, inner nuclear layer; IPL, inner plexiform layer; PHL, photoreceptor layer; OPL, outer plexiform layer; PE, pigmented epithelium. Scale bars = 25 μm.

For *crha*, labeling was absent in the zebrafish retina (Figure 4F), but the cichlid retina contained scattered *crha*-expressing cells in the amacrine cell region of the INL (Figures 4I, 5). These *crha*-expressing cells were less numerous than the *crhb*-expressing cells found in this same layer, and double fluorescent *in situ* hybridization showed that *crha* and *crhb* are not co-localized to the same cells (Figure 5).

### *crh* localization in the brain

**Figure 6:**
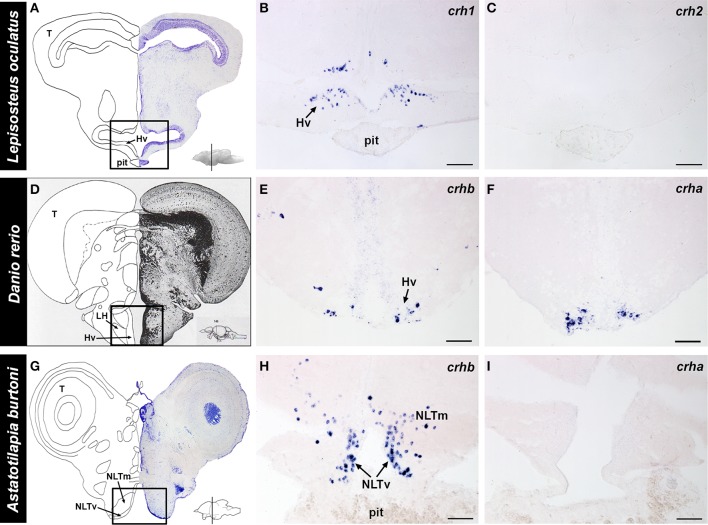
**Localization of *crh* forms in the hypothalamus of fishes**. **(A)** Representative transverse section of the spotted gar (*Lepisosteus oculatus*) brain stained with cresyl violet, with the approximate region of interest indicated (black box). **(B)** *crh1*-expressing cells were abundant in the ventral hypothalamus (Hv) of the gar, in the region above the pituitary (pit). **(C)** *crh2* expression was absent from the hypothalamus of the spotted gar. **(D)** Representative transverse section from the zebrafish (*Danio rerio*) brain atlas (Wullimann et al., 1996), with the region of interest indicated (black box). **(E)** Zebrafish *crhb*-expressing cells were found in the nucleus lateralis tuberis (NLT) (labeled ventral hypothalamus, Hv, in atlas), but were also widely distributed throughout the brain. **(F)** Zebrafish *crha* expression was found exclusively in the NLT, and nowhere else in the brain. **(G)** Representative transverse section of the cichlid (*Astatotilapia burtoni*) brain stained with cresyl violet, with the approximate region of interest indicated (black box). **(H)** *crhb*-expressing cells were abundant in the NLT region above the pituitary gland of the cichlid. **(I)** *crha* expression was not detectable in the cichlid brain. Insets on **(A,D,G)** show lateral view of the brain with approximate location of transverse sections indicated (line). For each species, panels are adjacent sections labeled with different anti-sense *crh* probes. Hv, ventral zone of the periventricular hypothalamus; LH, lateral hypothalamic nucleus; NLTm, medial part of lateral tuberal nucleus; NLTv, ventral part of lateral tuberal nucleus; pit, pituitary; T, tectum. Scale bars = 100 μm **(B,C)**; 25 μm **(E,F,H,I)**.

The complete localization of *crh1* and *crh2* in the spotted gar brain was described previously (Grone and Maruska, 2015). Briefly, in the spotted gar, *crh1* is widely distributed throughout the brain, including the ventral hypothalamus (Figure 6). In contrast, *crh2*-expressing cells are absent from the hypothalamus and in fact, are restricted to the secondary gustatory/visceral nucleus of the hindbrain.

Because the distribution of *crhb* in zebrafish and *A. burtoni* brains has been previously described (Alderman and Bernier, 2007, 2009; Chandrasekar et al., 2007; Chen and Fernald, 2008), our focus in the current report was to describe the expression patterns of *crha* in these teleosts. In the zebrafish, *crha*-expressing cells were stained strongly and specifically and were restricted to the lateral tuberal nucleus in the ventral hypothalamus (Figure 6). In *A. burtoni*, there was no *crha* labeling observed anywhere in the brain.

## Discussion

Our results provide evidence that (1) teleosts have two distinct corticotropin-releasing hormone genes, *crha* and *crhb*, (2) these two genes evolved via a teleost-specific duplication of *crh1*, (3) the ancestral fish *crh1* gene paralog found in the spotted gar is expressed in both retina and brain, and (4) *crha* has been differentially restricted to either retina or brain in *Astatotilapia burtoni* and *Danio rerio*, respectively.

### Phylogenetics

**Figure 7:**
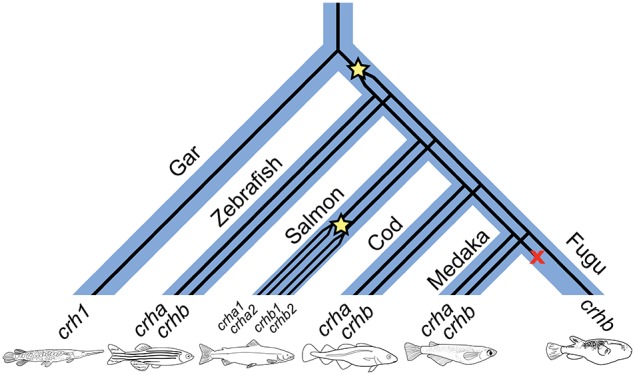
**A proposed history of CRH gene duplications and losses in teleost fishes based on our phylogenetic analyses**. Wide blue lines represent diverging species lineages; narrow black lines represent the CRH gene lineage. We propose that *crha* and *crhb* genes originated in early teleosts from a duplication (indicated by star in the figure) of a *crh1* gene homologous to the *crh1* that is found in extant non-teleost vertebrates including the spotted gar. Paralogs of *crha* and *crhb* are found in many teleost lineages, including cyprinids (e.g., zebrafish), gadiformes (e.g., Atlantic cod), and beloniformes (e.g., medaka). Salmoniformes (e.g., Atlantic salmon) experienced an additional genome duplication and retain two duplicates of both *crha* and *crhb*. Because *crha* homologs are not found in fugu genome sequence, we propose that *crha* has been lost in this lineage (indicated by an X in the figure).

We examined a broad range of the available teleost sequences for *crha* and *crhb* genes. Our findings of conserved synteny and conserved sequence across teleost species argue strongly for an origin of the *crha* and *crhb* genes in the teleost-specific whole genome duplication (Figure 7). Our synteny and phylogenetic analyses combine to clearly show that *crha* genes in the species we examined are more similar to each other than to *crhb* genes, despite their higher levels of sequence variability. Our phylogenetic analyses were not able to restrict all Crha protein sequences into a well-defined clade, most likely because of the relatively poor sequence conservation of Crha compared to Crhb (Figure 1). Zebrafish, the only ostariophysan species in our tree, is distantly related to the other teleosts, which is reflected in the relatively large branch lengths of both zebrafish Crha and Crhb. Because Crha and Crhb protein sequences are short and highly variable, it is not surprising that phylogenetic relationships reconstructed using only these proteins do not perfectly match known teleost species relationships.

Our identification of *crh1* and *crh2* as ohnologs likely arising from the second whole-genome duplication (WG2) suggested that Urotensin 1 (*uts1*) and a proto-*CRH* gene originated in the first whole-genome duplication (WG1) (Grone and Maruska, 2015), overturning the previously established assumption that *uts1* and *crh* did not diverge until WG2 (Lovejoy and de Lannoy, 2013). Similarly, the elucidation of teleost *crha* and *crhb* clarifies some previous misconceptions about the evolutionary origins of teleost crh genes. Some teleost lineages, including salmoniformes and cypriniformes such as carps, have experienced additional genome duplications subsequent to the third one (WG3) common to all teleosts (Ohno et al., 1968; David et al., 2003; Braasch and Postlethwait, 2012). Many of these teleosts, including salmonid and carp species, have duplicated *crhb* genes (Flik et al., 2006), which were sometimes misunderstood to have arisen in the teleost whole-genome duplication (Alsop and Vijayan, 2009). The very high degree of sequence identity between these duplicated *crhb* genes now appears less surprising, as it is apparent that their origins lie in the more recent lineage-specific genome duplications rather than WG3.

In a recent paper attempting phylogenetic analyses of CRH genes in teleosts, the diverse sequences of *crha* and *crhb* genes in teleosts led to misidentification of medaka *crha* as a separate gene distinct from both *crha* and *crhb* (Hosono et al., 2015). Although Hosono et al. mention that *crha* had been identified in zebrafish, they did not include zebrafish *crha* and *crhb* in their phylogeny. Hosono et al. also did not take into account the reduplication of CRH genes in salmonids. Our more detailed look at duplicated CRH genes in fishes has revealed that medaka *crha* is homologous to zebrafish and other teleost *crha* genes (see Figures 1–3 of the current report, and Grone and Maruska, 2015). The available data suggest that two teleost CRH genes, *crha* and *crhb*, exist in many teleosts but have been differentially lost or reduplicated in some teleost lineages. A separate teleost CRH gene, which Hosono et al. called “teleocortin,” does not exist.

Interestingly, Hosono et al. found that the predicted medaka *crha* peptide was a potent *in vitro* activator of both CRH receptors (Hosono et al., 2015). They reported high levels of expression of medaka *crha* mRNA in the retina, and *crha* mRNA was also detected in several other tissues, including the brain. Their data support the idea that *crha* may have different functions across teleost species.

Analyses of fossil records and phylogenetic evidence point to an origin for teleosts about 300 million years ago, during the Carboniferous to early Permian geological era (Near et al., 2012). Following the teleost WGD, many genes including neuropeptides were retained in two copies in teleosts, and have evolved differential expression (Glasauer and Neuhauss, 2014). For example the two ohnologs of pro-opiomelanocortin (*pomca* and *pomcb*), which encode adrenocorticotropic hormone (ACTH) as well as other peptides, were differentially restricted to preoptic area and pituitary, respectively, in *Tetraodon nigroviridis* (de Souza et al., 2005).

The potential for a wide range of outcomes for genes following WGD has been supported by theoretical considerations as well as genome-wide studies in fishes (Brunet et al., 2006; Kassahn et al., 2009). Following gene duplications, including those caused by whole-genome duplication, genes may experience non-functionalization, subfunctionalization, or neofunctionalization. Subfunctionalization may happen via protein changes (Hughes, 1994) or via regulatory element loss (Force et al., 1999) as described by the “duplication-degeneration-complementation” model, in which ancestral expression domains are differentially lost in different genes (Lynch and Force, 2000). Rapid subfunctionalization may be accompanied by prolonged neofunctionalization, as described by the “subneofunctionalization” model (He and Zhang, 2005). Teleost fish genomes exhibit diverse outcomes following whole genome duplication, as seen, for example, in *pax6* genes (Nornes et al., 1998), *mitf* genes (Lister et al., 2001), homeobox genes (Mungpakdee et al., 2008), and circadian clock genes (Toloza-Villalobos et al., 2015).

It is intriguing that *crh2* has been lost in both teleosts and eutherian mammals, two groups with very successful adaptive radiations in very different environments (Hoegg et al., 2004; Bininda-Emonds et al., 2007). Although we found *crh2* mRNA in the spotted gar hindbrain, it is not clear what the expression pattern (or functions) of *crh2* in a hypothetical teleost ancestor would have been. One possibility is that, early in teleost evolution, the presence of *crha* and *crhb* provided sufficient opportunity for multiple specialized functions of the CRH gene family, which could have allowed *crh2* to be lost.

### CRH in the retina

The expression of *crha* in *Astatotilapia burtoni* retina is a striking difference from the absence of *crha* in zebrafish retina. The retinal expression of *crha* could be a general feature of Perciform fishes, or might be a derived feature of the clade that includes *A. burtoni*. Similarly, we do not yet know if retinal *crha* is universally absent in Cypriniforms, or is a derived feature of zebrafish. *A. burtoni* is known as a “highly visual” cichlid species, communicating social information with colorful visual signals (Fernald and Hirata, 1979) as part of a more complex multisensory repertoire (Maruska and Fernald, 2012; Maruska et al., 2012). Previous studies suggest that male-male visual information is sufficient to change physiology and behavior in this species (Chen and Fernald, 2011). In this context, neuromodulation via Crha in the retina could facilitate signal processing before it ever reaches the brain.

There were also distinct differences in retinal *crhb* expression domains between zebrafish and *A. burtoni*: amacrine and ganglion cell layers of zebrafish, and amacrine and bipolar cells of *A. burtoni*. To our knowledge, this is the first evidence of *crh* expression in bipolar cells of any species. CRH and CRH receptors, however, are found in the retina of many species (Kiyama et al., 1984; Skofitsch and Jacobowitz, 1984; Williamson and Eldred, 1989; Olianas and Onali, 1995), suggesting that CRH signaling has an important retinal neuromodulatory role across all vertebrates. In the retina, both Crha and Crhb might act to mediate social information or stress responses in context-specific or species-specific manners, or might serve other unrelated and yet undiscovered roles.

The primitive neopterygian gars (Lepisosteiformes) belong to the infraclass Holostei that is considered an outgroup to the teleosts (Broughton et al., 2013). Lepisosteiformes are a particularly valuable group for understanding mechanisms of vertebrate molecular evolution, thanks to their ecological and evolutionary proximity to teleosts. Although gars are not identical to the ancestors of teleost fishes, their expression of *crh1* in the retina is likely to reflect the ancestral state, since other vertebrates including tetrapods have expression of *CRH1* in the retina as well as broadly in the brain (Kiyama et al., 1984; Skofitsch and Jacobowitz, 1984; Williamson and Eldred, 1989).

### CRH in the brain

Many immunohistochemical studies in teleost brains have found CRH-like immunoreactivity that likely corresponds largely to Crhb peptides, (e.g., Batten et al., 1990; Zupanc et al., 1999; Pepels et al., 2002). Teleost *crhb* genes are broadly expressed in the brain. Expression of zebrafish *crhb* in the brain has been documented via *in situ* hybridization in adults (Alderman and Bernier, 2007) and larvae (Chandrasekar et al., 2007). In *A. burtoni, crhb* is also expressed in many brain regions, including hypothalamus (Chen and Fernald, 2008; Carpenter et al., 2014). In zebrafish, one region of *crhb* mRNA expression is throughout the periventricular hypothalamus, in both caudal (Hc) and ventral (Hv) zones (Alderman and Bernier, 2007). The hypothalamic region referred to in many fish as nucleus lateralis tuberis (NLT) includes the equivalent of zebrafish ventral zone of periventricular hypothalamus (Hv) and lateral hypothalamic nucleus (LH) (Wullimann et al., 1996; Cerdá-Reverter et al., 2001; Zmora et al., 2014). Lesions in the goldfish NLT impaired corticosteroid release in response to stressors (Fryer and Peter, 1977), suggesting a key role for NLT in controlling pituitary adrenocorticotropic hormone (ACTH) release.

Zebrafish Hv or NLT is considered homologous to the mammalian arcuate nucleus (Song et al., 2003; Kurrasch et al., 2009). This region has important roles in nutrient metabolism, feeding, and neuroendocrine regulation. Agouti-related peptide (AGRP) and pro-opiomelanocortin (POMC) mRNA transcripts are found in zebrafish Hv neurons that project to the pituitary (Zhang et al., 2012). The connectivity and molecular identity of the *crha*-expressing neurons in zebrafish are unknown. Given the known importance of CRH peptides and the Hv nucleus in controlling food intake in fishes, *crha* neurons in the zebrafish Hv could be involved in regulation of feeding behavior as well as locomotor activity, anxiety, stress-coping, and other behaviors (Backström and Winberg, 2013; Matsuda, 2013).

## Conclusions

Powerful genomic resources for diverse fishes, including many teleosts as well as spotted gar, allowed us to examine evolution of ohnologs that arose from whole-genome duplications. Because many neuroanatomical structures, including retina and brain nuclei, are highly conserved, we were also able to compare the diversity of expression domains with precision. Our results highlight the fact that retention of a given ohnolog in multiple lineages does not demonstrate retention of the same function. Different lineages may experience differential regulatory element loss or gain as well as differential gene loss. Studying teleost *crha* and *crhb* genes will deepen our understanding of evolution and function of vertebrate neuropeptide signaling systems. We anticipate that interesting future comparisons will be made between the restricted roles of *crh2* in non-teleost vertebrates and *crha* in teleosts. By examining the restricted or novel functions of duplicated CRH genes, we will come to a greater understanding of CRH neuron development, anatomy, activity, and function. Furthermore, the restriction of *crha* to the retina in a highly visual species (*A. burtoni*) but not in another teleost (*D. rerio*) suggests a more general model in which duplicated neuropeptide genes may evolve expression domains that reflect the different ecological and behavioral specializations of different species.

The two teleost species we examined had divergent expression profiles for *crha*. Teleost superorders Ostariophysi (including zebrafish) and Acanthopterygii (including cichlids, medaka, sticklebacks, and pufferfish) diverged early in teleost evolution. Ostariophysi and Acanthopterygii have lost different ohnologs for many genes (Garcia de la Serrana et al., 2014). Our data are not sufficient to show at what point in evolutionary history the expression of *crha* was restricted to the retina in cichlids and to the hypothalamus in zebrafish. Furthermore, it is possible that *crha* may have additional or complementary expression domains in other teleosts. Future comparative work will be necessary to reveal the functional diversity of *crha* and *crhb* genes.

## Author contributions

Both authors had full access to all of the data in the study and take responsibility for its collection and analysis. Study concept and design: BG, KM. Acquisition of data: BG, KM. Analysis and interpretation of data: BG, KM. Drafting of the manuscript: BG, KM. Critical revision of the manuscript for important intellectual content: KM, BG. Obtained funding: KM. Administrative, technical, and material support: KM.

## Funding

This work was supported by startup funds from the College of Science and Department of Biological Sciences at Louisiana State University, a Ralph E. Powe Faculty Enhancement Award from Oak Ridge Associated Universities, and a Louisiana Board of Regents Research Competitiveness Subprogram Grant.

### Conflict of interest statement

The authors declare that the research was conducted in the absence of any commercial or financial relationships that could be construed as a potential conflict of interest.

## References

[B1] Abi-Rached L. Gilles A. Shiina T. Pontarotti P. Inoko H. (2002). Evidence of en bloc duplication in vertebrate genomes. Nat. Genet. 31, 100–105. 10.1038/ng855 11967531

[B2] Alderman S. L. Bernier N. J. (2007). Localization of corticotropin-releasing factor, urotensin I, and CRF-binding protein gene expression in the brain of the zebrafish, Danio rerio. J. Comp. Neurol. 502, 783–793. 10.1002/cne.21332 17436299

[B3] Alderman S. L. Bernier N. J. (2009). Ontogeny of the corticotropin-releasing factor system in zebrafish. Gen. Comp. Endocrinol. 164, 61–69. 10.1016/j.ygcen.2009.04.007 19366623

[B4] Alsop D. Vijayan M. (2009). The zebrafish stress axis: molecular fallout from the teleost-specific genome duplication event. Gen. Comp. Endocrinol. 161, 62–66. 10.1016/j.ygcen.2008.09.011 18930731

[B5] Amores A. Catchen J. Ferrara A. Fontenot Q. Postlethwait J. H. (2011). Genome evolution and meiotic maps by massively parallel DNA sequencing: spotted gar, an outgroup for the teleost genome duplication. Genetics 188, 799–808. 10.1534/genetics.111.127324 21828280 PMC3176089

[B6] Backström T. Winberg S. (2013). Central corticotropin releasing factor and social stress. Front. Neurosci. 7:117. 10.3389/fnins.2013.00117 23847465 PMC3705187

[B7] Batten T. F. Cambre M. L. Moons L. Vandesande F. (1990). Comparative distribution of neuropeptide-immunoreactive systems in the brain of the green molly, Poecilia latipinna. J. Comp. Neurol. 302, 893–919. 10.1002/cne.903020416 2081820

[B8] Berthelot C. Brunet F. Chalopin D. Juanchich A. Bernard M. Noël B.. (2014). The rainbow trout genome provides novel insights into evolution after whole-genome duplication in vertebrates. Nat. Commun. 5, 3657. 10.1038/ncomms4657 24755649 PMC4071752

[B9] Bininda-Emonds O. R. Cardillo M. Jones K. E. MacPhee R. D. Beck R. M. Grenyer R.. (2007). The delayed rise of present-day mammals. Nature 446, 507–512. 10.1038/nature05634 17392779

[B10] Braasch I. Peterson S. M. Desvignes T. McCluskey B. M. Batzel P. Postlethwait J. H. (2015). A new model army: emerging fish models to study the genomics of vertebrate Evo-Devo. J. Exp. Zool. Part B Mol. Dev. Evol. 324, 316–341. 10.1002/jez.b.22589 25111899 PMC4324401

[B11] Braasch I. Postlethwait J. (2012). Polyploidy in fish and the teleost genome duplication, in Polyploidy and Genome Evolution, eds Soltis P. S. Soltis D. E. (Berlin; New York, NY: Springer Verlag), 341–383.

[B12] Brawand D. Wagner C. E. Li Y. I. Malinsky M. Keller I. Fan S.. (2014). The genomic substrate for adaptive radiation in African cichlid fish. Nature 513, 375–381. 10.1038/nature13726 25186727 PMC4353498

[B13] Broughton R. E. Betancur R. R. Li C. Arratia G. Ortí G. (2013). Multi-locus phylogenetic analysis reveals the pattern and tempo of bony fish evolution. PLoS Curr. 5:ecurrents.tol.2ca8041495ffafd0c92756e75247483e. 10.1371/currents.tol.2ca8041495ffafd0c92756e75247483e 23788273 PMC3682800

[B14] Brunet F. G. Roest Crollius H. Paris M. Aury J. M. Gibert P. Jaillon O.. (2006). Gene loss and evolutionary rates following whole-genome duplication in teleost fishes. Mol. Biol. Evol. 23, 1808–1816. 10.1093/molbev/msl049 16809621

[B15] Burge C. Karlin S. (1997). Prediction of complete gene structures in human genomic DNA. J. Mol. Biol. 268, 78–94. 10.1006/jmbi.1997.0951 9149143

[B16] Carpenter R. E. Maruska K. P. Becker L. Fernald R. D. (2014). Social opportunity rapidly regulates expression of CRF and CRF receptors in the brain during social ascent of a teleost fish, Astatotilapia burtoni. PLoS ONE 9:e96632. 10.1371/journal.pone.0096632 24824619 PMC4019471

[B17] Cerdá-Reverter J. M. Zanuy S. Muñoz-Cueto J. A. (2001). Cytoarchitectonic study of the brain of a perciform species, the sea bass (Dicentrarchus labrax). II. The diencephalon. J. Morphol. 247, 229–251. 10.1002/1097-4687(200103)247:3<229::AID-JMOR1014>3.0.CO;2-K 11223930

[B18] Chandrasekar G. Lauter G. Hauptmann G. (2007). Distribution of corticotropin-releasing hormone in the developing zebrafish brain. J. Comp. Neurol. 505, 337–351. 10.1002/cne.21496 17912740

[B19] Chen C. C. Fernald R. D. (2008). Sequences, expression patterns and regulation of the corticotropin-releasing factor system in a teleost. Gen. Comp. Endocrinol. 157, 148–155. 10.1016/j.ygcen.2008.04.003 18501902 PMC3357958

[B20] Chen C. C. Fernald R. D. (2011). Visual information alone changes behavior and physiology during social interactions in a cichlid fish (Astatotilapia burtoni). PLoS ONE 6:e20313. 10.1371/journal.pone.0020313 21633515 PMC3102105

[B21] Christoffels A. Koh E. G. Chia J. M. Brenner S. Aparicio S. Venkatesh B. (2004). Fugu genome analysis provides evidence for a whole-genome duplication early during the evolution of ray-finned fishes. Mol. Biol. Evol. 21, 1146–1151. 10.1093/molbev/msh114 15014147

[B22] David L. Blum S. Feldman M. W. Lavi U. Hillel J. (2003). Recent duplication of the common carp (Cyprinus carpio L.) genome as revealed by analyses of microsatellite loci. Mol. Biol. Evol. 20, 1425–1434. 10.1093/molbev/msg173 12832638

[B23] Dehal P. Boore J. L. (2005). Two rounds of whole genome duplication in the ancestral vertebrate. PLoS Biol. 3:e314. 10.1371/journal.pbio.0030314 16128622 PMC1197285

[B24] de Souza F. S. Bumaschny V. F. Low M. J. Rubinstein M. (2005). Subfunctionalization of expression and peptide domains following the ancient duplication of the proopiomelanocortin gene in teleost fishes. Mol. Biol. Evol. 22, 2417–2427. 10.1093/molbev/msi236 16093565

[B25] Fernald R. D. (1977). Quantitative behavioural observations of Haplochromis burtoni under semi-natural conditions. Anim. Behav. 25, 643–653. 10.1016/0003-3472(77)90115-4

[B26] Fernald R. D. Hirata N. R. (1979). The ontogeny of social behavior and body coloration in the African cichlid fish Haplochromis burtoni. Zeitschrift für Tierpsychologie 50, 180–187.

[B27] Flik G. Klaren P. H. Van Den Burg E. H. Metz J. R. Huising M. O. (2006). CRF and stress in fish. Gen. Comp. Endocrinol. 146, 36–44. 10.1016/j.ygcen.2005.11.005 16403502

[B28] Force A. Lynch M. Pickett F. B. Amores A. Yan Y. L. Postlethwait J. (1999). Preservation of duplicate genes by complementary, degenerative mutations. Genetics 151, 1531–1545. 10101175 10.1093/genetics/151.4.1531 PMC1460548

[B29] Fryer J. N. Peter R. E. (1977). Hypothalamic control ACTH secretion in goldfish: II. Hypothalamic lesioning studies. Gen. Comp. Endocrinol. 33, 202–214. 10.1016/0016-6480(77)90245-3 200520

[B30] Garcia De La Serrana D. Mareco E. A. Johnston I. A. (2014). Systematic variation in the pattern of gene paralog retention between the teleost superorders Ostariophysi and Acanthopterygii. Genome Biol. Evol. 6, 981–987. 10.1093/gbe/evu074 24732281 PMC4007551

[B31] Gehrke A. R. Schneider I. de la Calle-Mustienes E. Tena J. J. Gomez-Marin C. Chandran M.. (2015). Deep conservation of wrist and digit enhancers in fish. Proc. Natl. Acad. Sci. U.S.A. 112, 803–808. 10.1073/pnas.1420208112 25535365 PMC4311833

[B32] Glasauer S. M. Neuhauss S. C. (2014). Whole-genome duplication in teleost fishes and its evolutionary consequences. Mol. Gen. Genom. 289, 1045–1060. 10.1007/s00438-014-0889-2 25092473

[B33] Grone B. P. Maruska K. P. (2015). A second corticotropin-releasing hormone gene (CRH2) is conserved across vertebrate classes and expressed in the hindbrain of a basal neopterygian fish, the spotted gar (Lepisosteus oculatus). J. Comp. Neurol. 523, 1125–1143. 10.1002/cne.23729 25521515

[B34] Guindon S. Gascuel O. (2003). A simple, fast, and accurate algorithm to estimate large phylogenies by maximum likelihood. Syst. Biol. 52, 696–704. 10.1080/10635150390235520 14530136

[B35] Hauger R. L. Grigoriadis D. E. Dallman M. F. Plotsky P. M. Vale W. W. Dautzenberg F. M. (2003). International union of pharmacology. XXXVI. Current status of the nomenclature for receptors for corticotropin-releasing factor and their ligands. Pharmacol. Rev. 55, 21–26. 10.1124/pr.55.1.3 12615952

[B36] He X. Zhang J. (2005). Rapid subfunctionalization accompanied by prolonged and substantial neofunctionalization in duplicate gene evolution. Genetics 169, 1157–1164. 10.1534/genetics.104.037051 15654095 PMC1449125

[B37] Hoegg S. Brinkmann H. Taylor J. S. Meyer A. (2004). Phylogenetic timing of the fish-specific genome duplication correlates with the diversification of teleost fish. J. Mol. Evol. 59, 190–203. 10.1007/s00239-004-2613-z 15486693

[B38] Hosono K. Kikuchi Y. Miyanishi H. Hiraki-Kajiyama T. Takeuchi A. Nakasone K. (2015). Teleocortin: a novel member of the corticotropin-releasing hormone family in teleost fish. Endocrinology 156, 2949–2957. 10.1210/en.2015-1042 26030477

[B39] Howe K. Clark M. D. Torroja C. F. Torrance J. Berthelot C. Muffato M.. (2013). The zebrafish reference genome sequence and its relationship to the human genome. Nature 496, 498–503. 10.1038/nature12111 23594743 PMC3703927

[B40] Hughes A. L. (1994). The evolution of functionally novel proteins after gene duplication. Proc. Biol. Sci. 256, 119–124. 10.1098/rspb.1994.0058 8029240

[B41] Jaillon O. Aury J. M. Brunet F. Petit J. L. Stange-Thomann N. Mauceli E.. (2004). Genome duplication in the teleost fish Tetraodon nigroviridis reveals the early vertebrate proto-karyotype. Nature 431, 946–957. 10.1038/nature03025 15496914

[B42] Jones D. T. Taylor W. R. Thornton J. M. (1992). The rapid generation of mutation data matrices from protein sequences. Comp. Appl. Biosci. 8, 275–282. 10.1093/bioinformatics/8.3.275 1633570

[B43] Jones F. C. Grabherr M. G. Chan Y. F. Russell P. Mauceli E. Johnson J.. (2012). The genomic basis of adaptive evolution in threespine sticklebacks. Nature 484, 55–61. 10.1038/nature10944 22481358 PMC3322419

[B44] Kasahara M. Naruse K. Sasaki S. Nakatani Y. Qu W. Ahsan B.. (2007). The medaka draft genome and insights into vertebrate genome evolution. Nature 447, 714–719. 10.1038/nature05846 17554307

[B45] Kassahn K. S. Dang V. T. Wilkins S. J. Perkins A. C. Ragan M. A. (2009). Evolution of gene function and regulatory control after whole-genome duplication: comparative analyses in vertebrates. Genome Res. 19, 1404–1418. 10.1101/gr.086827.108 19439512 PMC2720184

[B46] Katoh K. Standley D. M. (2013). MAFFT multiple sequence alignment software version 7: improvements in performance and usability. Mol. Biol. Evol. 30, 772–780. 10.1093/molbev/mst010 23329690 PMC3603318

[B47] Kearse M. Moir R. Wilson A. Stones-Havas S. Cheung M. Sturrock S.. (2012). Geneious Basic: an integrated and extendable desktop software platform for the organization and analysis of sequence data. Bioinformatics 28, 1647–1649. 10.1093/bioinformatics/bts199 22543367 PMC3371832

[B48] Kiyama H. Shiosaka S. Kuwayama Y. Shibasaki T. Ling N. Tohyama M. (1984). Corticotropin-releasing factor in the amacrine cells of the chicken retina. Brain Res. 298, 197–200. 10.1016/0006-8993(84)91170-3 6372944

[B49] Korosi A. Baram T. Z. (2008). The central corticotropin releasing factor system during development and adulthood. Eur. J. Pharmacol. 583, 204–214. 10.1016/j.ejphar.2007.11.066 18275957 PMC2329668

[B50] Kovács K. J. (2013). CRH: the link between hormonal-, metabolic- and behavioral responses to stress. J. Chem. Neuroanat. 54, 25–33. 10.1016/j.jchemneu.2013.05.003 23774011

[B51] Kurrasch D. M. Nevin L. M. Wong J. S. Baier H. Ingraham H. A. (2009). Neuroendocrine transcriptional programs adapt dynamically to the supply and demand for neuropeptides as revealed in NSF mutant zebrafish. Neural Dev. 4:22. 10.1186/1749-8104-4-22 19549326 PMC2715394

[B52] Lister J. A. Close J. Raible D. W. (2001). Duplicate mitf genes in zebrafish: complementary expression and conservation of melanogenic potential. Dev. Biol. 237, 333–344. 10.1006/dbio.2001.0379 11543618

[B53] Louis A. Muffato M. Roest Crollius H. (2013). Genomicus: five genome browsers for comparative genomics in eukaryota. Nucleic Acids Res. 41, D700–D705. 10.1093/nar/gks1156 23193262 PMC3531091

[B54] Lovejoy D. A. Chang B. S. Lovejoy N. R. del Castillo J. (2014). Molecular evolution of GPCRs: CRH/CRH receptors. J. Mol. Endocrinol. 52, T43–T60. 10.1530/JME-13-0238 24711645

[B55] Lovejoy D. A. de Lannoy L. (2013). Evolution and phylogeny of the corticotropin-releasing factor (CRF) family of peptides: expansion and specialization in the vertebrates. J. Chem. Neuroanat. 54, 50–56. 10.1016/j.jchemneu.2013.09.006 24076419

[B56] Lynch M. Force A. (2000). The probability of duplicate gene preservation by subfunctionalization. Genetics 154, 459–473. 10629003 10.1093/genetics/154.1.459 PMC1460895

[B57] Maruska K. P. Fernald R. D. (2012). Contextual chemosensory urine signaling in an African cichlid fish. J. Exp. Biol. 215, 68–74. 10.1242/jeb.062794 22162854 PMC3233390

[B58] Maruska K. P. Ung U. S. Fernald R. D. (2012). The African cichlid fish Astatotilapia burtoni uses acoustic communication for reproduction: sound production, hearing, and behavioral significance. PLoS ONE 7:e37612. 10.1371/journal.pone.0037612 22624055 PMC3356291

[B59] Matsuda K. (2013). Regulation of feeding behavior and psychomotor activity by corticotropin-releasing hormone (CRH) in fish. Front. Neurosci. 7:91. 10.3389/fnins.2013.00091 23754974 PMC3667241

[B60] Mungpakdee S. Seo H. C. Angotzi A. R. Dong X. Akalin A. Chourrout D. (2008). Differential evolution of the 13 Atlantic salmon Hox clusters. Mol. Biol. Evol. 25, 1333–1343. 10.1093/molbev/msn097 18424774

[B61] Near T. J. Eytan R. I. Dornburg A. Kuhn K. L. Moore J. A. Davis M. P.. (2012). Resolution of ray-finned fish phylogeny and timing of diversification. Proc. Natl. Acad. Sci. U.S.A. 109, 13698–13703. 10.1073/pnas.1206625109 22869754 PMC3427055

[B62] Nock T. G. Chand D. Lovejoy D. A. (2011). Identification of members of the gonadotropin-releasing hormone (GnRH), corticotropin-releasing factor (CRF) families in the genome of the holocephalan, *Callorhinchus milii* (elephant shark). Gen. Comp. Endocrinol. 171, 237–244. 10.1016/j.ygcen.2011.02.001 21310155

[B63] Nornes S. Clarkson M. Mikkola I. Pedersen M. Bardsley A. Martinez J. P.. (1998). Zebrafish contains two pax6 genes involved in eye development. Mech. Dev. 77, 185–196. 10.1016/S0925-4773(98)00156-7 9831649

[B64] Ohno S. (1970). Evolution by Gene Duplication. Berlin; New York, NY: Springer-Verlag.

[B65] Ohno S. Wolf U. Atkin N. B. (1968). Evolution from fish to mammals by gene duplication. Hereditas 59, 169–187. 10.1111/j.1601-5223.1968.tb02169.x 5662632

[B66] Olianas M. C. Onali P. (1995). G protein-coupled corticotropin-releasing hormone receptors in rat retina. Regul. Pept. 56, 61–70. 10.1016/0167-0115(95)00007-X 7770634

[B67] Pepels P. P. Meek J. Wendelaar Bonga S. E. Balm P. H. (2002). Distribution and quantification of corticotropin-releasing hormone (CRH) in the brain of the teleost fish Oreochromis mossambicus (tilapia). J. Comp. Neurol. 453, 247–268. 10.1002/cne.10377 12378586

[B68] Skofitsch G. Jacobowitz D. M. (1984). Corticotropin releasing factor-like immunoreactive neurons in the rat retina. Brain Res. Bull. 12, 539–542. 10.1016/0361-9230(84)90169-2 6380651

[B69] Song Y. Golling G. Thacker T. L. Cone R. D. (2003). Agouti-related protein (AGRP) is conserved and regulated by metabolic state in the zebrafish, Danio rerio. Endocrine 22, 257–265. 10.1385/ENDO:22:3:257 14709799

[B70] Star B. Nederbragt A. J. Jentoft S. Grimholt U. Malmstrøm M. Gregers T. F.. (2011). The genome sequence of Atlantic cod reveals a unique immune system. Nature 477, 207–210. 10.1038/nature10342 21832995 PMC3537168

[B71] Swanson L. W. Sawchenko P. E. Rivier J. Vale W. W. (1983). Organization of ovine corticotropin-releasing factor immunoreactive cells and fibers in the rat brain: an immunohistochemical study. Neuroendocrinology 36, 165–186. 10.1159/000123454 6601247

[B72] Tamura K. Peterson D. Peterson N. Stecher G. Nei M. Kumar S. (2011). MEGA5: molecular evolutionary genetics analysis using maximum likelihood, evolutionary distance, and maximum parsimony methods. Mol. Biol. Evol. 28, 2731–2739. 10.1093/molbev/msr121 21546353 PMC3203626

[B73] Toloza-Villalobos J. Arroyo J. I. Opazo J. C. (2015). The circadian clock of teleost fish: a comparative analysis reveals distinct fates for duplicated genes. J. Mol. Evol. 80, 57–64. 10.1007/s00239-014-9660-x 25487517

[B74] Vale W. Spiess J. Rivier C. Rivier J. (1981). Characterization of a 41-residue ovine hypothalamic peptide that stimulates secretion of corticotropin and beta-endorphin. Science 213, 1394–1397. 10.1126/science.6267699 6267699

[B75] Williamson D. E. Eldred W. D. (1989). Amacrine and ganglion cells with corticotropin-releasing-factor-like immunoreactivity in the turtle retina. J. Comp. Neurol. 280, 424–435. 10.1002/cne.902800308 2783937

[B76] Wullimann M. F. Rupp B. Reichert H. (1996). Neuroanatomy of the Zebrafish Brain: A Topological Atlas. Basel; Boston, MA: Birkhäuser Verlag.

[B77] Zhang C. Forlano P. M. Cone R. D. (2012). AgRP and POMC neurons are hypophysiotropic and coordinately regulate multiple endocrine axes in a larval teleost. Cell Metab. 15, 256–264. 10.1016/j.cmet.2011.12.014 22245570 PMC3332529

[B78] Zmora N. Stubblefield J. Golan M. Servili A. Levavi-Sivan B. Zohar Y. (2014). The medio-basal hypothalamus as a dynamic and plastic reproduction-related kisspeptin-gnrh-pituitary center in fish. Endocrinology 155, 1874–1886. 10.1210/en.2013-1894 24484170

[B79] Zupanc G. K. Horschke I. Lovejoy D. A. (1999). Corticotropin releasing factor in the brain of the gymnotiform fish, Apteronotus leptorhynchus: immunohistochemical studies combined with neuronal tract tracing. Gen. Comp. Endocrinol. 114, 349–364. 10.1006/gcen.1999.7273 10336823

